# Development and Evaluation of Enzyme-Linked Viral Immune Capture Assay for Detection of SARS-CoV-2

**DOI:** 10.3389/fbioe.2022.898726

**Published:** 2022-08-08

**Authors:** Naif Khalaf Alharbi, Nosaibah Samman, Sadeem Alhayli, Majed F. Alghoribi, Abdulrahman Almasoud, Atef Nehdi

**Affiliations:** ^1^ King Abdullah International Medical Research Center (KAIMRC), Riyadh, Saudi Arabia; ^2^ King Saud Bin Abdulaziz University for Health Science (KSAU-HS), Riyadh, Saudi Arabia; ^3^ Department of Life Sciences, Faculty of Sciences of Gabes, University of Gabes, Gabes, Tunisia

**Keywords:** SARS-CoV-2, detection, diagnosis, ELISA, immune capture

## Abstract

The pandemic of COVID-19 was caused by the severe acute respiratory syndrome coronavirus 2 (SARS-CoV-2) in 2019 and it has prompted unprecedented research activities for vaccines, therapeutics, and diagnostics. The real-time reverse transcriptase-polymerase chain reaction (RT-PCR) is the gold standard method of diagnosis; however, immune-based assays offer cost-effective, deployable, easy-to-read solutions for diagnosis and surveillance. Here, we present the development, optimization, and testing of an enzyme-linked viral immune capture assay (ELVICA). It utilizes the spike antigen as the detected target of the virus and antibody-coated beads to capture the virus and enrich the detection. This method can be readout by luminescent and colorimetric equipment. It can also be visualized by the imaging system, offering a variety of detection approaches. ELVICA showed specificity to SARS-CoV-2-pseudotyped viruses as compared to MERS-CoV-pseudotyped viruses. As compared to RT-PCR, ELVICA showed high compatibility in detecting the virus in patient respiratory samples, especially for samples that are below a Ct value of 32 in RT-PCR. This assay is readily adaptable for detecting other pathogens and serves as a quick and affordable diagnostic tool.

## Introduction

The emergence of the severe acute respiratory syndrome coronavirus 2 (SARS-CoV-2) in 2019 has quickly caused a global pandemic with around 200 million cases reported to the WHO and a mortality rate of 2% (World Health Organization, 2020). The virus causes the coronavirus infectious disease 2019 (COVID-19) by infecting the upper and lower respiratory tract cells in humans via the cellular receptor angiotensin-converting enzyme 2 (ACE2) (Lan et al., 2020). This leads to the rapid transmission of the virus, resulting in quick spread of the pandemic in different parts of the world, which warrants quick and effective measures. Although there are several approved vaccines and monoclonal antibody therapeutics, the virus continues to cause outbreaks and requires pharmaceutical and public health interventions. Therefore, diagnosing the viral infection early in symptomatic patients or asymptomatic individuals is an essential part of controlling the pandemic.

The reverse transcriptase-polymerase chain reaction (RT-PCR) is the gold standard method of laboratory diagnosis. This, however, requires laborious techniques, trained technicians, advanced instruments, cost, and time. Several other options have been tried to complement or substitute RT-PCR such as techniques that focused on detecting the viral particles or parts (proteins) of the viral particles. These options are mainly based on immunological principle of detecting viral antigens by the provision of anti-virus antibodies. For examples, lateral flow immunological assays and rapid antigen tests have been developed by a number of biotechnology companies (Hashem et al., 2020; Ernst et al., 2021; Hayer et al., 2021; Kevadiya et al., 2021). The principle of such tests is that the antigen–antibody complex will be visualized by adding a colorimetric substrate to enable the result to be read instantly by the eyes. However, these tests may not show high sensitivity or specificity rates that vary across suppliers; the sensitivity and specificity of ELISA are usually lower than RT-PCR (Mohit et al., 2021). ELISA is considered the most reliable immunoassays, which are usually developed and validated commercially and approved by regulatory agencies such as the FDA. Several ELISAs are now used in diagnostic labs; however, the majority of them are aimed at detecting anti-SARS-CoV-2 antibodies in sera rather than being a diagnostic tool *per se*. In addition, some ELISA tests have limitations of false-positive or false-negative rates (Chauhan et al., 2021a). Taken together, it remains important to optimize, or develop, new immunoassay technologies in order to improve the sensitivity and specificity of immunological assays as well as to have point-of-need, cost-effective, and fast diagnostics tool for COVID-19 as well as to accelerate immunogenicity studies of vaccines that are under development (Chauhan et al., 2021b).

Here, this study presents an optimized ELISA method that employs antigen capture and sample enrichment in order to increase the detection limit and enhance sensitivity. The method is named enzyme-linked viral immune capture assay (ELVICA) and utilizes magnetic nanoparticle beads to enrich the sample prior to the development of the test by chemical substrate. Although capture ELISA has been used in many diagnostic ELISA kits (Young et al., 2000), the utilization of magnetic beads has only been described previously for antibody detection in sera (Huergo et al., 2021) and employs different strategies of beads (Wu et al., 2020). This study presents testing of human nasal swabs from healthy individuals and COVID-19 patients.

## Results

### Generation and Optimization of ELVICA

**FIGURE 1:**
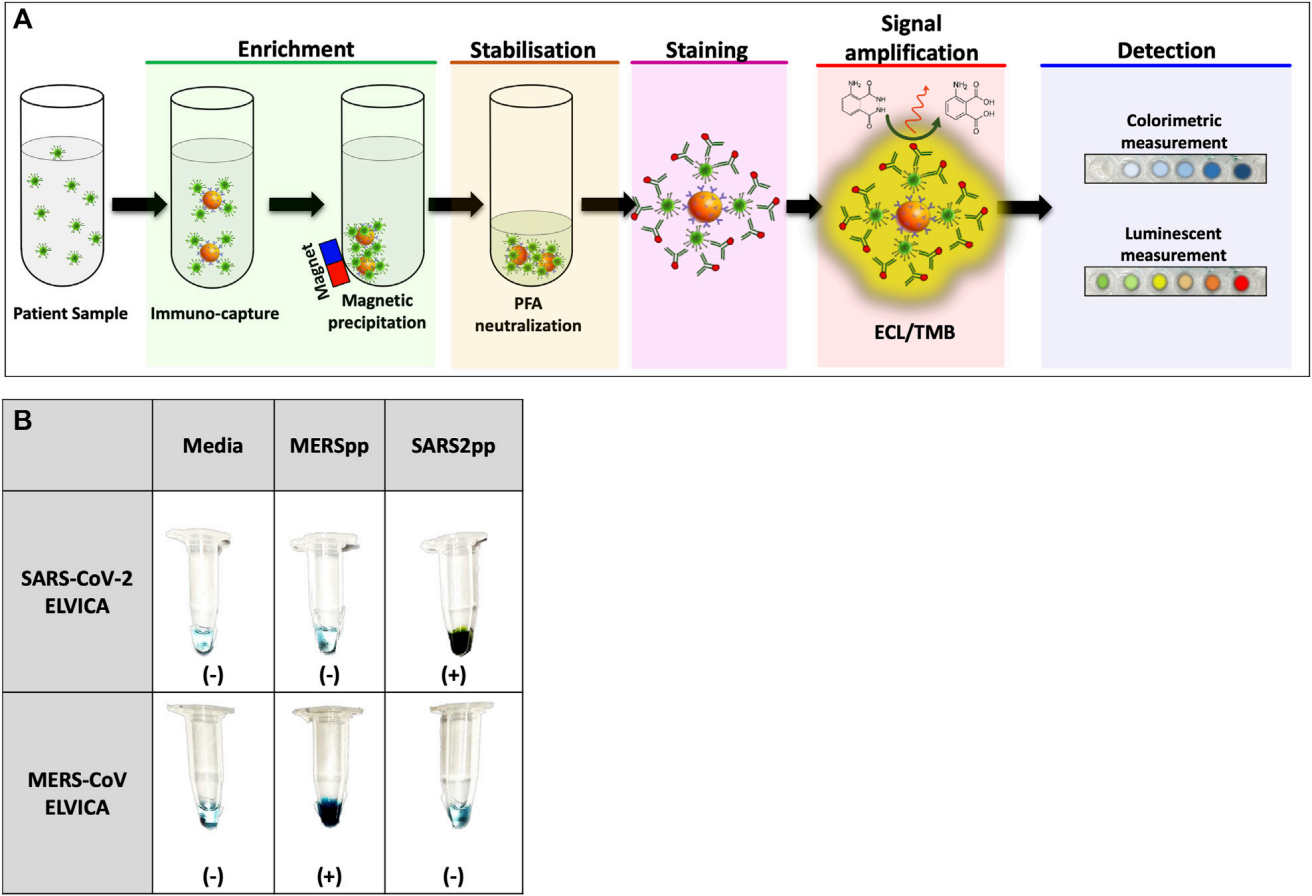
Representation of the sequential steps and visualization of ELVICA test. **(A)** ELVICA test consists of five consecutive steps: enrichment of viral particles by magnetic immune capture; stabilization of immune-captured viral particles with paraformaldehyde (PFA); staining of immune-captured viral particles with highly specific anti-spike antibody; development of signal amplification by ECL or TMB substrates of HRP; and signal detection using colorimetric or luminescent measurement. **(B)** Visualization of colorimetric ELVICA in tubes for SARS2pp and MERSpp; ELVICA was performed on media, SARSpp, or MERSpp. HRP: horse radish peroxidase. ECL: enhanced chemiluminescence substrate. TMB: 3,3′,5,5′-tetramethylbenzidine.

ELVICA was developed as an assay to detect SARS-CoV-2 spike protein and it involves five sequential steps, Figure 1. First (enrichment step), viral particles in patient samples were enriched using magnetic immune capture. Magnetic nanoparticles were coated with specific anti-spike antibodies to bind to, and capture, the viral particles. Second, the captured viral particles are deactivated and the complexes of viral particle/magnetic nanoparticle are fixed using 4% paraformaldehyde. This is to stabilize the viral particle/magnetic nanoparticle complexes and deactivate the virus, allowing safe handling of samples. Third, staining of the stabilized complexes was achieved with highly specific anti-spike antibody conjugated with horse radish peroxidase (HRP). The HRP could be conjugated either to the primary capturing antibody or to the secondary detecting antibody. Fourth, the reaction was developed and signal was detected using colorimetric or luminescent methods. In colorimetric-based detection, HRP reacts with TMB substrate to produce a measurable color that correlates with the level of immunocaptured viral particles. In luminescent-based detection, HRP reacts with ECL substrate leading to light emission that is captured by a digital imaging system or a plate reader. Colorimetric ELVICA was performed in tubes to detect pseudoviral particles (pp) of SARS-CoV-2 (SARS2pp) or MERS-CoV (MERSpp). This allows options for labs with limited resources, Figure 1B.

### Specificity and Sensitivity of the ELVICA Test

**FIGURE 2:**
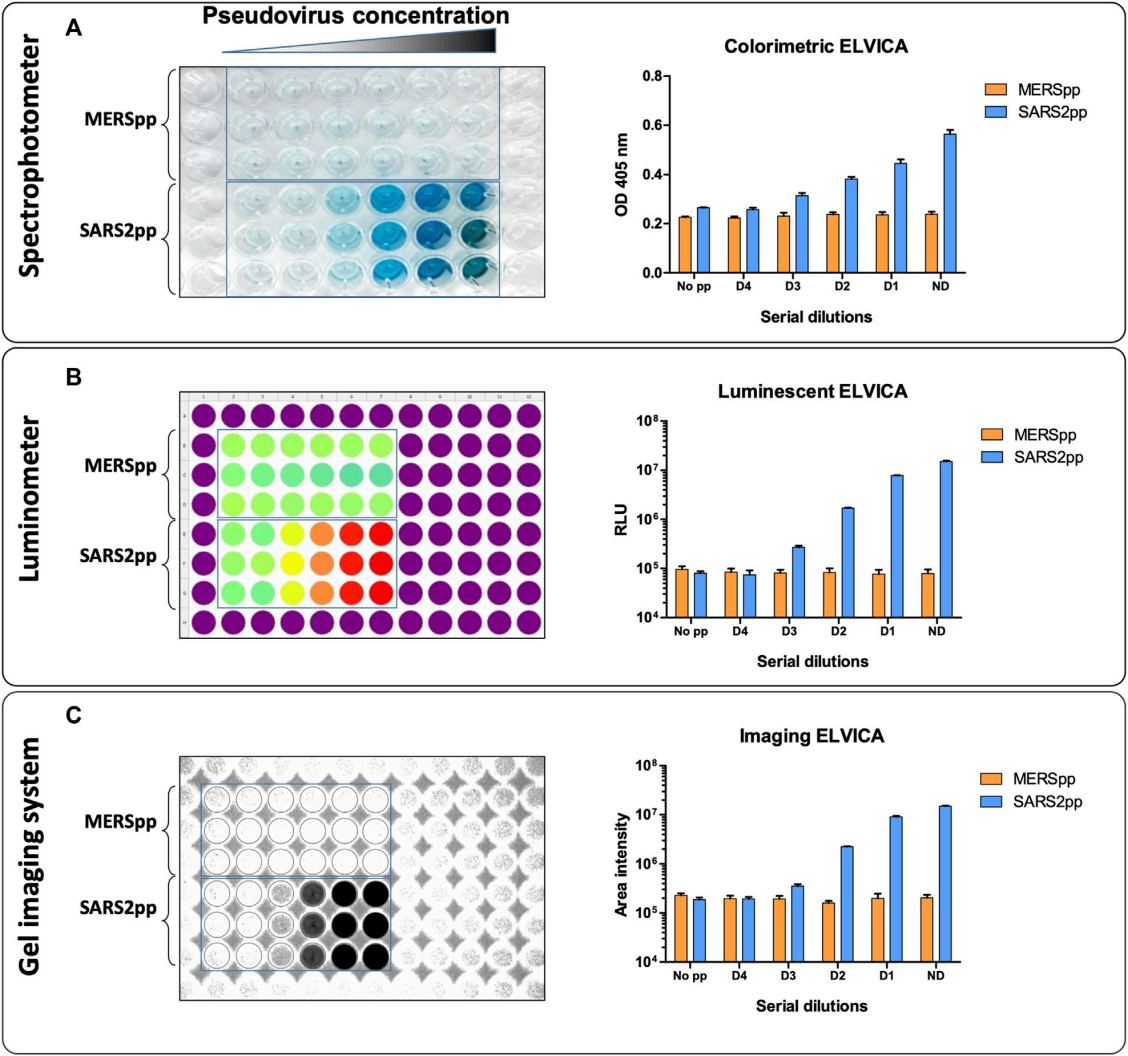
Detection of SARSpp and MERSpp in ELVICA. Colorimetric **(A)** and luminescent **(B, C)** detection using a spectrophotometer **(A)**, luminometer **(B)**, and digital gel imaging system **(C)** for the detection of SARS-CoV-2 pseudoviral particles (SARS2pp). MERSpp was used as a negative control.

**FIGURE 3:**
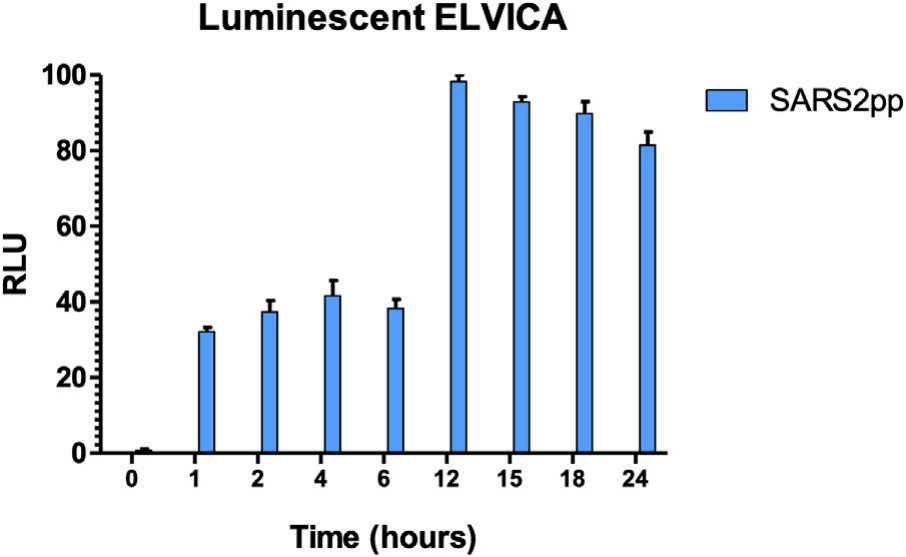
Time-dependent sensitivity of luminescent ELVICA for detecting SARS2pp. Time-dependent sensitivity of luminescent ELVICA performed at different incubation time periods with the same concentration of SARS2pp.

ELVICA was first evaluated for the detection of SARS2pp, and MERSpp was included as a negative control and to evaluate the specificity of the assay. Serial dilutions of SARS2pp starting from 10^5^ to 10^1^ of RLU/ml were tested in the assay using the two detection methods. Results showed that ELVICA is specific to SARS2pp as compared to MERSpp, there was a low level of non-specific background in these assays because this level was detected when no pseudoviruses were added in both detection methods, indicating that this background signal is a result of the assay, not the samples, Figure 2. The lowest detectable concentration of SARS2pp above the background level was 10^2^ RLU/ml; however, RLU is a relative measurement. In the luminescent detection method, results were obtained using a luminometer or a digital gel imaging system, and there was no difference in the level of detection, Figure 2. Overall, ELVICA shows strong specificity for SARS-CoV-2 in three different detection methods. The minimal incubation time required for optimal sensitivity of ELVICA in detecting SARS2pp was evaluated. From 1 to 6 hours of incubation achieved around 40% of detection. However, between 12 and 24 h of incubation, the detection outcomes were doubled. The optimal detection was 98% at 12 h of incubation, Figure 3.

### Detection of SARS-CoV-2 in Specimens From COVID-19 Patients

**FIGURE 4:**
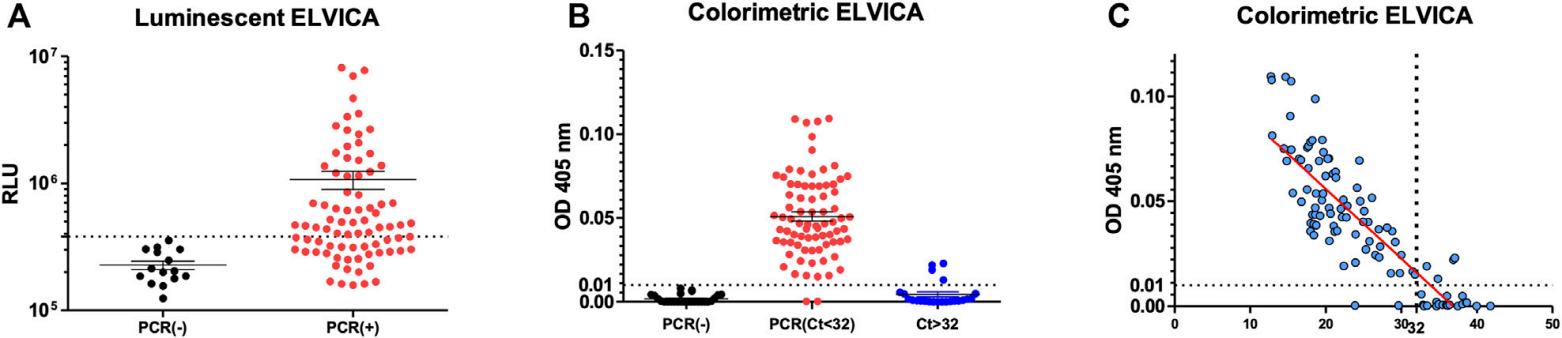
Detection of SARS-CoV-2 virus in human nasal swab samples using ELVICA. Detection of SARS-CoV-2 in PCR-positive and PCR-negative nasal swabs by luminescent ELVICA **(A)** or colorimetric ELVICA with additional samples based on the Ct values of RT-PCR **(B).** Correlation between colorimetric ELVICA results and PCR Ct values of the same nasal swab samples is shown in **(C)**. ELVICA limit of detection is shown as dotted lines.

ELVICA was performed to detect SARS-CoV-2 in nasal swabs from COVID-19 patients or non-COVID-19 patients. The samples were categorized as positive or negative as confirmed by SARS-CoV-2 diagnostic PCR. First, 90 samples were tested in luminescent ELVICA, of which 12 were PCR negative and 78 were PCR positive. PCR-negative samples showed a level of background signals in luminescent ELVICA that was 4.10^5^ RLU at the highest. The PCR-positive samples showed a range of RLUs that were around 10^7^ RLU at the highest. Although the mean RLU was higher in the PCR-positive samples by more than 1 log as compared to PCR-negative samples, there was an overlap in the RLU between some PCR-positive and PCR-negative samples in the luminescent ELVICA, which indicates a level of false-negative results in luminescent ELVICA (Figure 4A).

In order to circumvent the false-negative issue, the samples with additional 32 samples were categorized as: PCR negative, PCR positive (Ct values below 32), and PCR weak positive (Ct values above 32), and were tested in colorimetric ELVICA. The colorimetric detection and the cut-off of Ct < 32 showed improved detection of SARS-CoV-2 with false-negative rate of 2.4%. When all samples were considered regardless of the Ct values, false-negative rate was 17.4% (Figure 2B and Table 1). In addition, to confirm that the Ct value cut-off would be distinctive, correlation curve of ELVICA results in comparison to PCR Ct values determined the theoretic limit of detection (LOD) at Ct value of 32 (Figure 4C). Colorimetric ELVICA was used here because it showed a lower level of background signal and therefore might have higher specificity and could be useful in distinguishing positive samples. ELVICA results of samples that had Ct < 32 showed that only two false-negative samples were detected, Figure 4C.

**TABLE 1:** Comparison of false-negative and false-positive rates in colorimetric ELVICA for all samples (A) and samples with Ct < 32 (B). (+) and (-) indicate positive and negative results of PCR and ELVICA tests. Samples with Ct > 32 were considered negative in (B).

A: All samples		Colorimetric ELVICA	
		(+)	(−)
PCR	105 (+)	87 (82.8%)	18 (17.2%)
			False-negative
	12 (−)	0 0%	12 (100%)
		False-positive	
**B: Samples with Ct < 32**		**Colorimetric ELVICA**	
		**(+)**	**(−)**
PCR	84 (+)	82 (97.6%)	2 (2.4%)
			False-negative
	12(−) + 21 (ct > 32)	4 12.12%	29 (87.87%)
		False-positive	

Ct values are the readout of the RT-PCR diagnostic test in clinical labs

In order to set a potential reference range for ELVICA test, 12 of the 90 samples that were negative for SARS-CoV-2 by PCR were used to calculate the upper limit of the reference range. The calculation was based on a formula (see Method section) and showed that the upper limit of the reference range at 95% confidence interval was 378,538 (RLU) in luminescent ELVICA and 0.01 OD (absorbance at 405 nm) in colorimetric ELVICA.

## Discussion

In this study, we aimed to develop a novel immune capture assay, named enzyme-linked viral immune capture assay (ELVICA), for the detection of SARS-CoV-2. The utility of pseudoviral particles for both SARS-CoV-2 and MERS-CoV (SARS2pp and MERSpp) allowed quick handling of the assay in a biosafety-level two conditions for easy optimization of ELVICA. SARS2pp and MERSpp were produced using the same lentiviral system with the only difference being the spike protein of the viruses. ELVICA was specific for SARS2pp in comparison to MERSpp and *vice versa* when the assay was set up to detect MERSpp. It also showed a similar level of non-specific background with SARS2pp and MERSpp.

ELVICA results can be read by one of three options, spectrophotometry, luminescence readers, or gel documentation imaging system, with comparable results and similar lower limit of detection. All the three methods were able to detect SARS2pp down to a concentration of 10^2^ RLU/ml. The RLU/ml represents the unit of input inoculum used in ELVICA, which is an acceptable unit of quantifying luciferase-based pseudoviruses (Almasaud et al., 2020; Alserehi et al., 2020). The level of background between SARS2pp and MERSpp was similar in any of the three detection methods. Next, ELVICA was used to detect SARS-CoV-2 in human samples, previously confirmed by PCR to be negative or positive. Although the results of ELVICA showed an overlap between PCR negative and positive, raising a concern of false-negative results, colorimetric ELVICA showed improved detection as compared to the luminescent, which could be due to the limitation of utilized detection methods since high sensitivity of luminescence-based detection would lead to high background and subsequently higher number of false positive. The sensitivity of the different substrates used in the detection methods could also be a possible factor for higher false negative/positive (Chauhan et al., 2021a). Importantly, colorimetric ELVICA was distinct in detecting strong PCR-positive samples that had Ct value below 32. Therefore, colorimetric ELVICA can be useful for labs with limited resources in diagnosing suspected cases with symptoms and mild-to-high viral load.

Capture ELISA has been used in many diagnostic ELISA kits (Young et al., 2000); however, the utilization of magnetic beads has only been described previously for antibody detection in sera (Huergo et al., 2021) and this employed different strategies where beads were added to streptavidin not to capture antibody (Wu et al., 2020). Magnetic capture immune approaches offer strong diagnostic assays with ease of use in peripheral laboratories or labs with limited resources. It can be easily read by naked eyes giving a quick indication of SARS-CoV-2 infection. This gives it the potential to be used with minimal training for limited resource areas or labs. However, reliable, affordable, and quick (or point-of-care) serological assays are still required to encounter the pandemic by conducting efficient and real-time seroprevelance studies as well as to support immunogenicity testing of vaccines that are under development (Chauhan et al., 2021b). This would require global focus and ample funding (Chauhan et al., 2020).

Our current study has some limitations, including that the nasal swabs from COVID-19 patients had been tested fresh by PCR while they were tested in ELVICA after being frozen. A head-to-head comparison between PCR and ELVICA must be done in future analysis. Having fresh samples may increase the specificity of the ELVICA. In addition, PCR and ELVICA are testing two different determinants, RNA and spike protein, respectively, and the comparison was made with PCR because it is the gold standard method for SARS-CoV-2 detection. ELVICA has an extra enrichment step that magnifies antigen detection, unlike other antigen-based assays such as ELISA. ELVICA can also be utilized for research and surveillance purposes at low cost and with minimal technological requirements as opposed to molecular testing and PCR. It was successfully used to detect SARS-CoV-2 and MERS-CoV in this study, but it can be readily adaptable for detecting other viral and bacterial pathogens.

In conclusion, this study presents a novel immunological diagnostic assay for SARS-CoV-2 with encouraging results, especially with COVID-19 patient samples that have high viral load and Ct value below 32. The developed assay offers a range of possible readout methods making it suitable for less resourceful labs.

## Materials and Methods

### Patient Samples and Pseudoviral Particles

Nasopharyngeal human swab samples were obtained from the Ministry of National Guard Health Affairs (MNGHA) hospital, Riyadh, after the diagnostic PCR testing was performed, according to the established clinical diagnostic protocols (Young et al., 2021). All samples were frozen until the day of ELVICA test and were thawed once. Samples were handled as highly infectious according to safety guidelines established by MNGHA. MERS-CoV and SARS-CoV-2 pseudoviral particles were generated and quantified as relative light unit per ml (RLU/ml) following established protocols (Almasaud et al., 2020; Alserehi et al., 2020).

### ELVICA Test

For immune capture of SARS-CoV-2 particles, magnetic particles coated with anti-SARS-CoV-2 spike RBD rabbit polyclonal antibodies (MagIso™) (Creative Diagnostics, United States; cat # WHK-SN027) were utilized. SARS2pp or nasal swab samples were added in 500 ul to 10 ul of conjugated magnetic particles and incubated overnight rotating at room temperature. Next, the complexes were precipitated by magnets and fixed using 4% paraformaldehyde. The fixation step is followed by a washing step using wash buffer (20 mM Tris-HCl pH8, 137 mM NaCl, 5 mMKcl, 1 mM MgCl2, and 2 mM CaCl2) containing 0.1% (v/v) Tween three times. The complexes were then incubated with HRP-conjugated SARS-CoV-2 spike monoclonal Chimeric (rabbit variable region)/human (kappa/IgG1 constant) antibody (Sino Biological, China, Cat # 40,150-D006) at a dilution of 1% for 1 h. Following a three-time wash using wash buffer containing 0.3% Tween, signal was developed by resuspension of particles in either TMB (Cell Signaling) or ECL (Bio-Rad) substrates. Samples were transferred to 96-well plates and signal was detected. In colorimetric ELVICA, HRP reacts with TMB substrate to produce a measurable color at 405 nm (SpectraMax M5 spectrophotometer, Molecular devices, CA, United States). In luminescent ELVICA, HRP reacts with ECL substrate leading to light emission that is captured by a digital imaging system (Chemi-Doc Bio-Rad) or a luminometer (Multimode plate reader Envision, Perkin Elmer, MA, United States). Wash buffer was used as a negative control and SARS2pp as a positive control.

For immune capture of MERSpp, magnetic nanoparticles coated with anti-MERS-CoV spike protein S1, rabbit polyclonal antibody was used (Sino Biological, China, cat # 40,069-T52). For detection of MERSpp, anti-MERS-CoV spike protein S1 mouse monoclonal antibody conjugated with HRP was used (Sino Biological, China, cat # 40,069-MM23). The background upper limit at 95% confidence interval was calculated based on the formula:

Upper limit = average absorbance (RLU) of PCR confirmed negative samples + t0.975, n-1 X square root (n+1/n) * SD.

### Statistical Analysis

GraphPad Prism (GraphPad Software) was used for statistical analysis and to plot data.

## Data Availability

The original data presented in the study are included in the article/Supplementary Material; any further data inquiries can be directed to the corresponding author.

## References

[B1] Almasaud A. Alharbi N. K. Hashem A. M. (2020). Generation of MERS-CoV Pseudotyped Viral Particles for the Evaluation of Neutralizing Antibodies in Mammalian Sera. Methods Mol. Biol. 2099, 117–126. 10.1007/978-1-0716-0211-9_10 31883092 PMC7123069

[B2] Alserehi H. A. Alqunaibet A. M. Al-Tawfiq J. A. Alharbi N. K. Alshukairi A. N. Alanazi K. H. (2020). Seroprevalence of SARS-CoV-2 (COVID-19) Among Healthcare Workers in Saudi Arabia: Comparing Case and Control Hospitals. Diagn Microbiol. Infect. Dis. 99 (3), 115273. 10.1016/j.diagmicrobio.2020.115273 33296851 PMC7677039

[B3] Chauhan N. Soni S. Gupta A. Aslam M. Jain U. (2021). Interpretative Immune Targets and Contemporary Position for Vaccine Development against SARS‐CoV‐2: A Systematic Review. J. Med. Virol. 93 (4), 1967–1982. Available from: http://www.ncbi.nlm.nih.gov/pubmed/33270225 . 10.1002/jmv.26709 33270225 PMC7753271

[B4] Chauhan N. Soni S. Gupta A. Jain U. (2020). New and Developing Diagnostic Platforms for COVID-19: A Systematic Review. Expert Rev. Mol. Diagnostics 20 (9), 971–983. Available from: http://www.ncbi.nlm.nih.gov/pubmed/32896179 . 10.1080/14737159.2020.1816466 32896179

[B5] Chauhan N. Soni S. Jain U. (2021). Optimizing Testing Regimes for the Detection of COVID-19 in Children and Older Adults. Expert Rev. Mol. Diagnostics 21 (10), 999–1016. Available from: http://www.ncbi.nlm.nih.gov/pubmed/34324823 . 10.1080/14737159.2021.1962708 PMC8425447 34324823

[B6] Ernst E. Wolfe P. Stahura C. Edwards K. A. (2021). Technical Considerations to Development of Serological Tests for SARS-CoV-2. Talanta 224, 121883. [Internet]. Available from: http://www.ncbi.nlm.nih.gov/pubmed/33379092 . 10.1016/j.talanta.2020.121883 33379092 PMC7654332

[B7] Hashem A. M. Alhabbab R. Y. Algaissi A. Alfaleh M. A. Hala S. Abujamel T. S. (2020). Performance of Commercially Available Rapid Serological Assays for the Detection of SARS-CoV-2 Antibodies. Pathog 9 (12), 1067. [Internet]. Available from: http://www.ncbi.nlm.nih.gov/pubmed/33352788 . 10.3390/pathogens9121067 PMC7767212 33352788

[B8] Hayer J. Kasapic D. Zemmrich C. (2021). Real-world Clinical Performance of Commercial SARS-CoV-2 Rapid Antigen Tests in Suspected COVID-19: A Systematic Meta-Analysis of Available Data as of November 20, 2020. Int. J. Infect. Dis. 108, 592–602. [Internet]. Available from: http://www.ncbi.nlm.nih.gov/pubmed/34015523 . 10.1016/j.ijid.2021.05.029 34015523 PMC8127520

[B9] Huergo L. F. Selim K. A. Conzentino M. S. Gerhardt E. C. M. Santos A. R. S. Wagner B. (2021). Magnetic Bead-Based Immunoassay Allows Rapid, Inexpensive, and Quantitative Detection of Human SARS-CoV-2 Antibodies. ACS Sens. 6 (3), 703–708. Available from: http://www.ncbi.nlm.nih.gov/pubmed/33496577 . 10.1021/acssensors.0c02544 33496577

[B10] Kevadiya B. D. Machhi J. Herskovitz J. Oleynikov M. D. Blomberg W. R. Bajwa N. (2021). Diagnostics for SARS-CoV-2 Infections. Nat. Mat. 20 (5), 593–605. [Internet]. Available from: http://www.ncbi.nlm.nih.gov/pubmed/33589798 . 10.1038/s41563-020-00906-z PMC8264308 33589798

[B11] Lan J. Ge J. Yu J. Shan S. Zhou H. Fan S. (2020). Structure of the SARS-CoV-2 Spike Receptor-Binding Domain Bound to the ACE2 Receptor. Nature 581 (7807), 215–220. [Internet]. Available from: http://www.ncbi.nlm.nih.gov/pubmed/32225176 . 10.1038/s41586-020-2180-5 32225176

[B12] Mohit E. Rostami Z. Vahidi H. (2021). A Comparative Review of Immunoassays for COVID-19 Detection. Expert Rev. Clin. Immunol. 17 (6), 573–599. [Internet]. Available from: http://www.ncbi.nlm.nih.gov/pubmed/33787412. 10.1080/1744666x.2021.1908886 33787412

[B13] World Health Organisation (2020). Coronavirus Disease (COVID-19) Pandemic. [Internet]. Available from: https://www.who.int/emergencies/diseases/novel-coronavirus-2019 Accessed 10 July 2022.

[B14] Wu K. Saha R. Su D. Krishna V. D. Liu J. Cheeran M. C.-J. (2020). Magnetic-Nanosensor-Based Virus and Pathogen Detection Strategies before and during COVID-19. ACS Appl. Nano Mat. 3 (10), 9560–9580. 10.1021/acsanm.0c02048 37556271

[B15] Young B. E. Ong S. W. X. Ng L. F. P. Anderson D. E. Chia W. N. Chia P. Y. (2021). Viral Dynamics and Immune Correlates of COVID-19 Disease Severity. Clin Infect Dis Off Publ Infect Dis Soc Am 73 (9), e2932–e2942. 10.1093/cid/ciaa1280 PMC7499509 32856707

[B16] Young P. R. Hilditch P. A. Bletchly C. Halloran W. (2000). An Antigen Capture Enzyme-Linked Immunosorbent Assay Reveals High Levels of the Dengue Virus Protein NS1 in the Sera of Infected Patients. J. Clin. Microbiol. 38 (3), 1053–1057. Available from: http://www.ncbi.nlm.nih.gov/pubmed/10698995 . 10.1128/jcm.38.3.1053-1057.2000 10698995 PMC86336

